# Cardiovascular Assessments in Children and Adolescents With Hypertension

**DOI:** 10.31083/RCM39498

**Published:** 2025-08-25

**Authors:** Nafees Sathik, Rama Safadi, Ishveen Saini, Arsheya Ahuja, Jieji Hu, Rupesh Raina

**Affiliations:** ^1^College of Graduate Studies, Northeast Ohio Medical University, Rootstown, OH 44272, USA; ^2^College of Medicine, Northeast Ohio Medical University, Rootstown, OH 44272, USA; ^3^Department of Internal Medicine, Summa Health System, Akron, OH 44304, USA; ^4^Hathaway Brown School, Shaker Heights, OH 44122, USA; ^5^Department of Nephrology, Akron Nephrology Associates at Cleveland Clinic Akron General Medical Center, Akron, OH 44302, USA; ^6^Department of Pediatric Nephrology, Akron Children's Hospital, Akron, OH 44308, USA

**Keywords:** pediatric hypertension, arterial stiffness, ambulatory blood pressure monitoring, carotid intima-media thickness, left ventricular hypertrophy, pulse wave velocity, endothelial dysfunction

## Abstract

Cardiovascular assessments in children and adolescents with hypertension are essential for detecting early signs of organ damage and guiding timely interventions. The pathophysiology of pediatric hypertension involves a complex interplay of arterial stiffness, endothelial dysfunction, metabolic disturbances, activation of the renin–angiotensin–aldosterone system, and immune dysregulation. These mechanisms collectively contribute to target organ damage, particularly in the cardiovascular system. Traditional office-based blood pressure measurements often fail to identify individuals at high risk, prompting the adoption of more advanced diagnostic techniques. Measures of arterial stiffness, such as pulse wave velocity, augmentation index, and cardio–ankle vascular index, provide valuable insights into vascular health and have been strongly associated with left ventricular hypertrophy and impaired heart function. Imaging modalities, including carotid intima-media thickness and epicardial adipose tissue measurements, serve as indicators of subclinical atherosclerosis and cardiovascular risk. Advanced echocardiographic tools that assess myocardial strain and ventricular–arterial coupling provide a more nuanced understanding of cardiac performance in hypertensive adolescents. These advanced techniques enhance the early detection of cardiovascular abnormalities and support a more individualized approach to managing pediatric hypertension. However, challenges related to validation, standardization, and clinical integration remain. Thus, expanding access to these modalities and refining their use in pediatric populations are crucial steps toward improving long-term cardiovascular outcomes in youth with elevated blood pressure.

## 1. Introduction

Pediatric hypertension is an increasingly recognized clinical concern. In the United States, the prevalence of hypertension among children and adolescents is approximately 3.5%, while in Europe it is estimated at 5% [1]. Notably, the global prevalence of pediatric hypertension has increased significantly over the past two decades, with a reported increase of 75% to 79% between 2000 and 2015 [2]. Earlier diagnosis and treatment of hypertension, especially in children and adolescents, is important to improve health outcomes and quality of life in adulthood [3]. However, pediatricians often struggle to diagnose hypertension in children and adolescents, in part because there are no widely validated diagnostic values to guide accurate assessments [4]. One study reported that 71% of pediatricians routinely measure blood pressure during ambulatory visits, but only 65% of those physicians compare the readings to reference standards when elevated values are suspected [5, 6]. Among this subgroup, 47% misclassified elevated blood pressure as normotensive, despite meeting the diagnostic criteria for hypertension. Results from the Supporting Hypertension Awareness and Research Europe-wide (SHARE) survey concluded that physicians significantly underestimated the proportions of “challenging patients” with hypertension relative to their perceptions of the proportions of patients achieving European Society of Hypertension (ESH) targets for blood pressure [7].

Varying guidelines complicate the diagnosis of pediatric hypertension. The American Academy of Pediatrics (AAP) defines the threshold to be 130/80 mmHg for adolescents [8]. The ESH defines stage 1 hypertension as blood pressure between the 95th–99th percentile + 5 mmHg, while stage 2 hypertension is defined as blood pressure >99th percentile + 5 mmHg. For pediatric patients aged 16 years and older, high-normal blood pressure is considered to be in the range of 130–139 mmHg systolic and 85–89 mmHg diastolic, with high blood pressure defined as >140/90 mmHg [9]. There is a need for improved and validated methods for identifying hypertension in order to accurately diagnose hypertension and take timely precautions to deter the risks of untreated hypertension [10]. This review explores current and emerging cardiovascular assessment techniques that support the early diagnosis of hypertension and inform personalized management in children and adolescents.

## 2. Pathophysiology of Pediatric Hypertension

Factors that may influence the development of hypertension in children include vascular structure, mechanics and function, oxidative stress, hyperinsulinemia, insulin resistance, hyperlipidemia, renin-angiotensin-aldosterone elements, and immune abnormalities [11].

### 2.1 Vascular Structure, Mechanics, and Function

Macrocirculation modifications include arterial wall remodeling, hypertensive remodeling of carotid arteries, and disturbed serum concentrations of matrix metalloproteinases (MMPs) and their tissue inhibitors (TIMPs). Arterial stiffening, defined as the decreased flexibility of arterial walls, is a precursor to elevated blood pressure and can develop early in life [12]. Increased arterial stiffness is often found in adolescents with pre-hypertension and exacerbates the risk of developing cardiovascular disease [13, 14]. Stiffer arteries require the heart to exert a greater force to circulate blood, subsequently damaging small arterioles, arteries, and organs rich in arterial connections [15]. Hypertensive adolescent males were also found to have elevated distributed serum concentrations of MMP9 and TIMP1 and elevated expression of those two genes in peripheral blood leukocytes [16, 17].

**Fig. 1.**
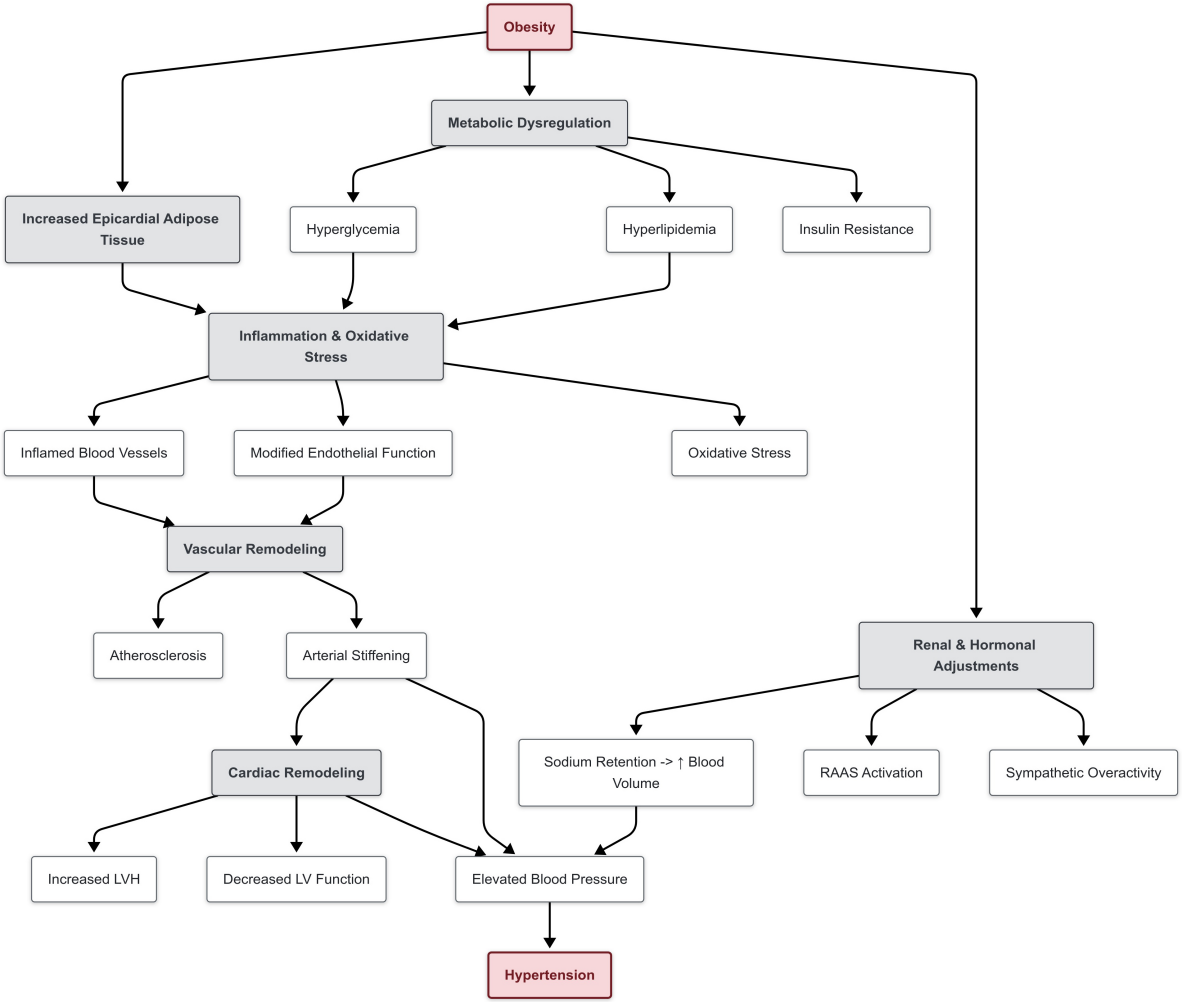
**Pathophysiology of hypertension in children**. ↑ Increase. Abbreviations: LV, left ventricle; LVH, left ventricular hypertrophy; RAAS, renin-angiotensin aldosterone system.

Microcirculation remodeling includes alterations to blood vessels via endothelial cells altercation. Endothelial cells are vital to the maintenance of cardiovascular health, as they have a key role in delivering oxygen and nutrients to other cells, regulating blood flow through constriction or dilation, modulating immune cell trafficking, and maintaining tissue homeostasis [18]. The dysfunction of those cells can be caused by cardiovascular risk factors such as hypertension, hyperlipidemia, and arterial inflammation. In coronary and peripheral arteries, these cardiovascular risk factors can lead to diminished endothelial integrity, increased expression of adhesion molecules, the release of cytokines, and the upregulation of antigen-presenting molecules. In combination, these effects characterize endothelial dysfunction and promote atherogenesis (Fig. 1) [19, 20]. Hypertensive patients have impaired endothelial-dependent vasodilation in both coronary arteries and the forearms, with arteries that are less reactive to stimulation [21, 22]. Finally, several studies utilize the narrowing of retinal vessels as a predictor of high blood pressure, arterial stiffening, and carotid stiffening [23, 24, 25].

### 2.2 Metabolic Abnormalities and Oxidative Stress

Children with primary hypertension are typically exposed to metabolic abnormalities, hyperinsulinemia, insulin resistance and oxidative stress [26]. These factors affect blood pressure as early as age four and are associated with modifying body composition [27]. As a result, obesity is a primary risk factor in developing hypertension. The mechanisms in which obesity causes hypertension are complex and include overactivation of the sympathetic nervous system (SNS), stimulation of the renin-angiotensin-aldosterone system, alteration in adipose-derived cytokines, insulin resistance, and structural and functional renal changes.

Pathophysiological mechanisms in which hyperinsulinemia can contribute to elevated blood pressure include the impairment of cell membrane ion-exchange, enhanced sympathetic and renin-angiotensin aldosterone system activation, sodium retention, volume expansion, left ventricular hypertrophy (LVH), and atherosclerosis [28]. Higher insulin levels and insulin resistance at the age of 13 are linked to both elevated blood pressure and hyperlipidemia by the age of 16 [29]. Impaired SNS activity is associated with elevated heart rate and blood pressure, and has also been linked to visceral obesity, metabolic abnormalities, and immune phenomena in pediatric patients with hypertension [12]. The results of the Bogalusa Heart Study associated increased heart rate with higher blood pressure and adiposity. As such, visceral obesity is a phenotypic feature of children with hypertension and can lead to greater sympathetic activity [13]. Oxidative stress, quantified by superoxide or oxidative stress markers, has also demonstrated to be elevated in children with primary hypertension irrespective of body mass index (BMI), while being correlated with hypertension mediated tissue damage [30].

Hyperlipidemia is also implicated in hypertension. Elevated levels of low-density lipoprotein (LDL)-cholesterol are a precursor for the development of atherosclerotic plaques. Continuous exposure to atherosclerotic plaques can lead to calcium accumulation in the coronary arteries, which increases the risk for ischemic heart disease in adulthood. Furthermore, atherosclerotic plaques can cause narrowing of arteries, requiring the heart to exert greater force, subsequently increasing arterial blood pressure [31].

### 2.3 Renin-Angiotensin-Aldosterone System

The renin-angiotensin aldosterone system (RAAS) contributes to hypertension through vasoconstriction, inflammation, and organ remodeling [32]. Hypertensive children have shown to have overactive RAAS [33]. Renin, when released in response to low blood pressure, converts angiotensinogen into angiotensin I, which is then converted into angiotensin II in the lungs. Angiotensin II vasoconstricts and stimulates the release of aldosterone, which promotes sodium and water retention, impacting blood volume, ultimately raising blood pressure [34]. Upregulation of this system, regardless of blood pressure, will lead to its elevation. Monogenic hypertension studies have also shown that salt-dependency is attributed to abnormalities in renin-angiotensin-aldosterone signaling. This can lead to sodium retention, increased blood volume, and subsequently elevated blood pressure [26, 35]. Furthermore, children who are salt sensitive have drastic increases in blood pressure due to their salt intake, while salt resistant children do not exhibit hypertension. Pediatric study has shown that salt sensitivity is evident in children with obesity, diabetes, and premature birth [26].

### 2.4 Immune Abnormalities

Hypertensive children may also have varied T-cell distribution relative to normotensive children [36, 37]. T-cell response creates a vicious cycle in primary hypertension, as arterial damage from elevated blood pressure generates autoantigens that further perpetuate hypertension as it further damages the arterial walls [38]. In addition, hypertensive children have shown significant activation of innate immunity with increased serum concentrations of C-reactive protein and chemokines, indicating endothelial activation [39]. Myeloid dendritic cells are more activated in children with white coat hypertension and in adolescents [40]. Additional immunological factors associated with elevated pediatric blood pressure include increased serum concentrations of C-reactive protein, chemokines, adiponectin, the *ras* genes, MMPs and TIMPs [26].

### 2.5 Hypertension Phenotypes

#### 2.5.1 Masked Hypertension 

Masked hypertension occurs when clinic blood pressure readings are normal, yet ambulatory blood pressure readings are abnormal [41]. Masked hypertension can be diagnosed on the premise of isolated and elevated wake or sleep (nocturnal) blood pressure, or a combination of both [41]. In patients with obesity, a history of repaired aortic coarctation, or chronic kidney disease (CKD), masked hypertension is more prevalent [41]. With the increased risk of masked hypertension, these patients also have a correlated increased left ventricular mass index, left ventricular hypertrophy, and pulse wave velocity [42]. In order to prevent potential progression to cardiovascular disease (CVD) in adulthood, hypertensive conditions must be identified and regularly monitored [41]. Ambulatory blood pressure monitoring (ABPM) may be used to enhance precision of screening and intervention in adolescents with elevated blood pressure, especially if masked [41].

#### 2.5.2 Nocturnal Hypertension 

Nocturnal hypertension, a form of circadian-associated masked hypertension, is common is patients with obesity, CKD, obstructive sleep apnea, systemic lupus erythematosus, and sickle cell disease, as well as in patients with previous solid-organ transplants [41]. Nocturnal hypertension can also manifest in patients with less severe conditions, including steroid-sensitive nephrotic syndrome, where 72% of pediatric patients have nocturnal hypertension [41]. In patients with CKD or diabetes, nocturnal hypertension is associated with increased carotid intima-media thickness (cIMT) and left ventricular hypertrophy (LVH) [41]. In patients with CKD or obesity, identifying nocturnal hypertension may aid in maximizing therapeutic benefits and reversing total organ damage [41].

#### 2.5.3 Isolated Systolic Hypertension 

Isolated systolic hypertension (ISH) is the most prevalent subset of hypertension in 12- to 16-year-olds in the United States [43]. ISH is influenced by increased elasticity of large vessels and can be attributed to the enhanced amplification of pulse pressure [43]. In pediatric patients with ISH, cIMT was found to be higher than those with normal blood pressures [43]. In comparing patients with ISH to those with systo-diastolic hypertension (SDH), those with ISH had relatively higher pulse pressure amplification, yet a significantly higher stroke volume [43]. This difference may be due to the increase in peripheral vascular resistance [43]. Elevated systolic blood pressure in adolescent years is associated with left ventricular hypertrophy later on in life [41]. Given this, early, targeted interventions in adolescents with ISH would be valuable [41].

#### 2.5.4 White Coat Hypertension 

White coat hypertension is present when clinical blood pressure readings are elevated, while ambulatory blood pressure readings are normal [41]. Although prevalence is unclear, white coat hypertension is widely observed [41]. Children with white coat hypertension are more associated with higher markers of cardiovascular disease and an elevated left ventricular in comparison to patients without white coat hypertension, yet lower than patients with ambulatory blood pressure [41]. Available data may suggest that white coat hypertension is an unclear phenotype, especially given the limited data available regarding management and long-term outcomes [41].

## 3. Clinical Assessment Tools for Hypertension 

### 3.1 Oscillometric Devices

Oscillometric devices are a more accurate measure of blood pressure, as they eliminate human error. These devices utilize proprietary algorithms specific to their manufacturers that associate the oscillations produced by the deflating cuff and the blood pressure measurement obtained [44]. Unlike auscultation with a sphygmomanometer, there are normative values that have been published for oscillometric devices, making it more favorable to utilize compared to auscultation [45].

### 3.2 Ambulatory Blood Pressure Monitoring

Beyond standard auscultation, the AAP recommends the usage of ABPM in all children who are suspected to have hypertension based on clinical blood pressure to confirm the diagnosis [8]. ABPM can also predict target organ damage due to hypertension and underlying cardiovascular health risks better than a clinical blood pressure reading [41, 46]. As ABPM monitors blood pressure over a 24-hour period, it reduces the impact of elevated blood pressure due to white coat hypertension (defined as clinical hypertension with normal ABPM) and enhances the diagnosis of masked hypertension (normal clinical blood pressure with hypertension on ABPM). Furthermore, ABPM can also be used to monitor nocturnal blood pressure, monitoring for the lack of the typical physiologic 10–20% decrease in blood pressure at night [47]. A lack of nocturnal blood pressure dipping may also be a strong independent predictor of cardiovascular events and mortality [48]. In 2022, the American Heart Association (AHA) eliminated the category of prehypertension and classified patients into white coat hypertension, masked hypertension, ambulatory hypertension, or normotension, while simultaneously lowering the nocturnal hypertension threshold to 110/65 mmHg [41]. Hill-Horowitz *et al*. [49] found that 70% of adolescents who were classified as prehypertensive prior to the change in guidelines are newly classified as hypertensive. Of those newly classified hypertensive adolescents, 57% of them had nocturnal hypertension as measured by ABPM [49].

### 3.3 Arterial Stiffness

Measuring arterial stiffness is useful to diagnose pediatric hypertension and can potentially detect subclinical inflammation [50]. In adolescents, the Hanzhong Adolescent Hypertension Study associated elevated childhood blood pressure and increased arterial stiffness (odds ratio (OR) = 1.69 (95% CI: 1.32–2.16)) [15, 51]. Cuspidi *et al*. [52] found that participants between ages 10–25 years who had arterial stiffening and LVH were at a twofold increased risk of masked hypertension (OR = 2.29 (95% CI: 1.01–5.31)). Furthermore, mean ambulatory systolic blood pressure was higher (mean difference +2.9 mmHg (95% CI: 0.4–5.3 mmHg, *p* < 0.05)), and there was a higher prevalence of nocturnal non-dipping of systolic blood pressure (SBP) (mean difference 3.2 mmHg (95% CI: 0.3–6.1 mmHg, *p* < 0.05)) [53]. Methods to measure arterial stiffening include pulse wave velocity (PWV), augmentation index, carotid-femoral PWV (cfPWV), and brachial artery pulse wave velocity (baPWV).

#### 3.3.1 Pulse Wave Velocity

PWV is the speed of the pressure waves released from cardiac contractions measured by Doppler ultrasound. Yang *et al*. [54] observed similar associations between elevated youth blood pressure and increased PWV (OR = 1.83), although the data incorporated in the study lacked baseline PWV measurements. Chung *et al*. [55] found that children with ambulatory hypertension were more at risk for elevated PWV (pooled difference = 0.39 m/s (95% CI: 0.20–0.58)). Similarly, utilizing ABPM, Kollios *et al*. [56] discovered that masked hypertension (4.72 m/s (95% CI: 4.62–4.82)) and sustained hypertension (4.79 m/s (95% CI: 4.65–4.94)) were independent predictors of elevated 24-hour PWV compared to normotensive participants (4.33 m/s (95% CI: 4.26–4.40)). When stratifying adolescent patients by systolic blood pressure, adolescents with high systolic blood pressure (SBP) (≥90th percentile, PWV = 5.35 (95% CI: 4.43–6.27)) also had increased arterial stiffness compared to moderate SBP (SBP ≥80th and <90th percentile, PWV = 5.08 (95% CI: 4.32–5.84)) and low SBP (SBP <75th percentile, PWV = 4.83 (95% CI: 4.14–5.52)) [57]. Given the established association between arterial stiffening and hypertension, the Polish Society of Hypertension and the European Society of Hypertension recommend that PWV ≥95th percentile for age and gender be considered evidence of hypertension-mediated organ damage [9, 58, 59].

#### 3.3.2 Augmentation Index

The augmentation index (Aix) measures the reflection of the pulse wave due to arterial rigidity. In flexible arteries, the wave returns sooner to the heart than in rigid arteries [15]. In children, Altay *et al*. [60] showed that Aix and central SBP had a significantly strong positive correlation (r = 0.88, *p* < 0.0001). Increased youth obesity has been linked with accelerated atherosclerosis and arterial stiffening, which increases the likelihood of being diagnosed with hypertension [12, 61]. Mihuta *et al*. [62] found that obese children had significantly higher PWV (median = 4.8 m/s) compared to the control group (median = 4.5 m/s). Similarly, children in the obese group (Aix = 29.28%) had a significantly (*p* < 0.03) higher Aix compared to the control group (Aix = 24.58%).

#### 3.3.3 Carotid Femoral Pulse Wave Velocity

The carotid-femoral PWV (cfPWV) measures the PWV specifically between the carotid artery and femoral artery. cfPWV has also been utilized to associate arterial stiffening with elevated blood pressure. In 3862 healthy adolescent patients, Agbaje [63] correlated higher cfPWV during adolescence with the risk of increased systolic BP (OR = 1.20 (95% CI: 1.02–1.41), *p* = 0.026) and elevated diastolic BP (OR = 1.77 (95% CI: 1.32–2.38), *p* < 0.0001). Stoner *et al*. [64] found an increase in cfPWV of 0.12 m/s (95% CI: 0.07–0.16 m/s) per year as well as a positive association between cfPWV and blood pressure. In 2023, Agbaje [65] conducted a prospective study of adolescents aged 17 and concluded that arterial stiffness served as a damping mechanism for the effects of hypertension, such as elevated blood pressure, and elevated LVH. Counterintuitively, arterial stiffness was associated with elevated diastolic blood pressure but lower left ventricular diastolic function [65].

#### 3.3.4 Cardio-Ankle Vascular Index

Similar to cfPWV, the cardio-ankle vascular index (CAVI) utilizes cardiac waves; however, CAVI measures PWV at the ankle. CAVI is considered a preferable measure of arterial stiffening, as it captures the majority of the ascending aorta, an area where early vascular stiffening most commonly develops [66, 67]. There have been mixed findings regarding CAVI and hypertension. In adolescents, Mestanik *et al*. [68] utilized CAVI to evaluate arterial stiffness and found there was a significant relationship between elevated CAVI, white coat hypertension, and essential hypertension. However, Harvey *et al*. [69] did not find any significant differences between CAVI values among normotensive children (CAVI = 4.94) and children with elevated blood pressure (CAVI = 5.12) or stage I/II hypertension (CAVI = 5.05). This suggests that CAVI is independent of blood pressure, which is likely not ideal for detecting hypertension but may identify early end-organ changes. Harvey *et al*. [69] did find a significant difference between adolescents with elevated left ventricular hypertrophy (LVH) and those who do not and their respective CAVI (LVH <48 g/m^2.7^; CAVI = 5.21 and LVH >48 g/m^2.7^; CAVI = 5.95, *p* = 0.018).

#### 3.3.5 Brachial Arterial Pulse Wave Velocity

Brachial arterial pulse wave velocity (baPWV) is another method of measuring arterial stiffness. It is calculated as the difference between the systolic and diastolic blood pressures at the brachial level. Arterial stiffening can serve as a predictor of hypertension in adolescents [63, 70]. Adults with persistently elevated systolic blood pressure (β = 5.358 (95% CI: 4.958–5.759), *p* < 0.001) had significantly increased baPWV compared to normotensive participants [12]. baPWV can also be used to independently predict the risk of hypertension and elevated systolic blood pressure upon clinic revisitation. Jiang *et al*. [71] stratified baPWV in Chinese children into four quartiles, with Q4 representing the highest values. Compared to those in Q1, children in Q4 exhibited a 2.72-fold increased risk of developing hypertension (95% CI: 1.54–4.78). The usage of baPWV can be utilized to differentiate children at high risk for hypertension and provide timely management.

#### 3.3.6 Additional Methods to Measure Arterial Stiffness

Arterial stiffness can also be imaged using a multidetector CT and MRI machine to detect perivascular lipid volume [72]. More recently, subclinical inflammation and arterial damage in children with primary hypertension monitored using the neutrophil-to-lymphocyte and platelet-to-lymphocyte ratios are promising potential biomarkers of arterial stiffness, requiring further research to validate their diagnostic value [50].

### 3.4 Cardiac Structure and Function

#### 3.4.1 Left Ventricular Hypertrophy and Function

Left ventricular size and function may be correlated with hypertension. McNiece *et al*. [73] demonstrated that children with stage 2 hypertension had a significantly greater prevalence of LVH compared to children with stage 1 hypertension, masked hypertension, white coat hypertension, and normal blood pressure (LVH prevalence = 32.4%, 18%, 22.2%, 9.4%, 5.7% respectively). The Hanzhong Adolescent Hypertension Study found that elevated childhood blood pressure was associated with LVH (OR: 1.86 (95% CI: 1.13–3.05)) [51]. Chung *et al*. [55] found that children with ambulatory hypertension were more at risk for LVH (OR: 4.69 (95% CI: 2.69–8.19)) compared to normotensive children.

Decreased left ventricular function due to LVH may be associated with hypertension and can be quantified using the E/e’ ratio on echocardiography (Table 1) [74]. Tran *et al*. [74] concluded children with a higher E/e’ ratio had higher risk for hypertension and for subclinical changes in left ventricular systolic and diastolic function. Children with hypertension and obesity have significant increases in the following metrics of left ventricular composition and function: left ventricular mass, relative wall thickness, end-diastolic internal diameter, diastolic interventricular septum thickness, diastolic posterior wall thickness, A peak, E’ peak, A’ peak, and E/e’ ratio [75]. Kloch-Badelek *et al*. [76], on behalf of the European Project on Genes in Hypertension (EPOGH) investigators, studied LV diastolic function in 1258 participants from a population based in Belgium, Italy, Poland, and Russia. The study found that 22–25% of European adults met the criteria for diastolic dysfunction and furthermore, those who had hypertension had significantly higher E/e’ ratios [76].

**Table 1.** **Diagnostic markers for the detection of cardiovascular damage hypertension**.

Technique	Imaging used	Function	Utility
Augmentation index	Echocardiography	Measures the reflection of the wave due to arterial rigidity	Arterial Stiffness
Cardio-ankle vascular index	Echocardiography	Measures pulse waves from the heart and the PWV is measured at the ankle	Arterial Stiffness
Carotid femoral PWV	Echocardiography	Measures pulse waves from the carotid and the PWV is measured at the femoral artery	Arterial Stiffness
Pulse wave velocity (PWV)	Doppler ultrasound	Measures the speed of pulse wave propagation	Arterial Stiffness
Common carotid artery measurements	Echocardiography	Measures carotid intima-media thickness	Carotid intima-media thickness
EAT volume	Echocardiography	Measures the amount of fat around the heart	Epicardial Adipose Tissue Thickness
A’ peak	Doppler ultrasound	Determines late diastolic filling of left ventricle	Left Ventricular Function
A peak	Doppler ultrasound	Determines late diastolic atrial contraction velocity in mitral inflow	LVH and Function
Diastolic interventricular septum thickness	Echocardiography	Measures thickness of interventricular septum in diastole	LVH and Function
Diastolic LV posterior wall thickness	Echocardiography	Measures thickness of left ventricular posterior wall at end-diastole	LVH and Function
E’ peak	Doppler ultrasound	Determines early diastolic myocardial relaxation at the mitral annulus	LVH and Function
E/e’ ratio	Doppler ultrasound	Measures left ventricular filling pressure and diastolic strain	LVH and Function
End-diastolic LV internal diameter	Echocardiography	Measures the size of left ventricular cavity at end-diastole	LVH and Function
LV mass (LVM)	Echocardiography	Measures total mass of left ventricle based on wall thickness and chamber size	LVH and Function
LVM index	Echocardiography	Normalizes left ventricular mass to body surface area	LVH and Function
Relative wall thickness	Echocardiography	Measures left ventricular remodeling by comparing the ratio of wall thickness and end-diastolic radius	LVH and Function
Ventricular arterial uncoupling	Echocardiography	Measures mismatch between ventricular contraction and arterial load	LVH and Function
Doppler renal sonography	Doppler ultrasound	Measures renal blood flow to detect vascular abnormalities	Renal Function

Abbreviations: LVH, left ventricular hypertrophy.

**Fig. 2.**
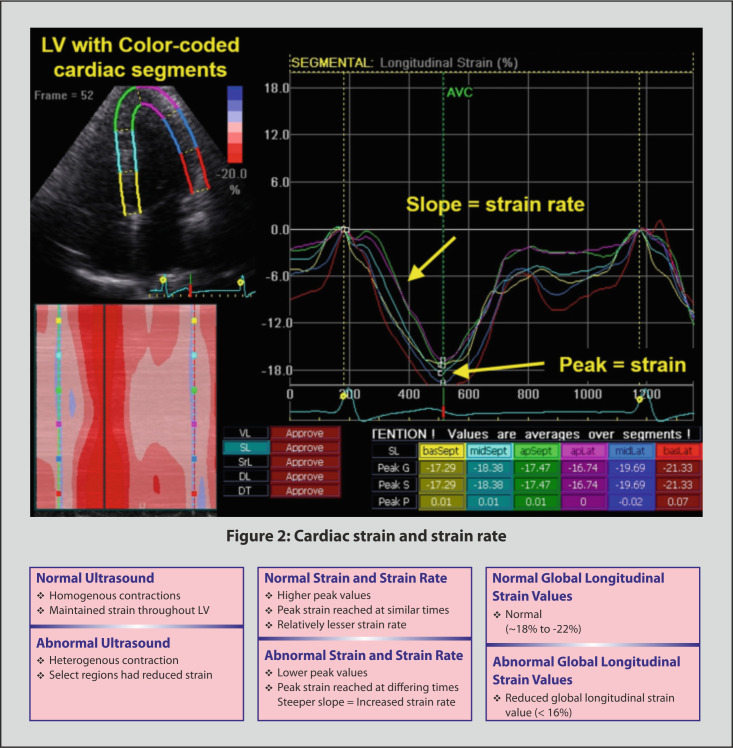
**Cardiac strain and strain rate [77, 78]**. Abbreviations: AVC, aortic valve closure; apSept, apical septal; apLat, apical lateral; basSept, basal septum; basLat, basal lateral; DL, diagonal longitudinal; DT, diagonal transverse; LV, left ventricle; midLat, mid lateral; SL, septal longitudinal; SrL, strain rate longitudinal; VL, ventricular longitudinal.

Cardiac strain assesses the left ventricular systolic function and can be measured with Doppler ultrasound to determine velocities of myocardial tissue movement. Additionally, speckle tracing is an ultrasound-based technique that tracks natural acoustic markers across frames, offering angle independence relative to tissue doppler imaging but with reduced resolution due to lower frame rate (Fig. 2, Ref. [77, 78]). Being able to measure the strain present in the left ventricle allows to detect the effort at which the heart pumps, with greater strain indicating greater risk for cardiovascular diseases such as hypertension [77].

Ventricular-arterial (VA) uncoupling is defined as the ratio of arterial elastance over ventricular elastance. If the ratio is greater than one, then there is uncoupling present, which has been implicated in impaired cardiac function [79]. Tso *et al*. [80] studied VA uncoupling in American college football players and determined that VA uncoupling is associated with increased systolic blood pressure and impairments to left ventricular function. Piskorz *et al*. [81] found that VA uncoupling was a predecessor to LVH and diastolic function, suggesting that VA uncoupling could be used to diagnose hypertension. Further study needs to be done in children to associate VA uncoupling with hypertension.

Suppression of tumorigenicity (ST2) is a biomarker of cardiac function and is released in the bloodstream when cardiomyocytes stretch [82]. Measuring levels of sST2 (soluable ST2) in the bloodstream can be utilized to determine LVH and subsequently be used to diagnose hypertension. Wang *et al*. [83] found a positive correlation between the concentrations of soluble venous ST2 and the prevalence of LVH (r = 0.315, *p* < 0.001). Wang *et al*. [83] also conducted a receiver operating characteristic analysis that resulted in sST2 having potential predictive value for LVH (AUC = 0.752 (95% CI: 0.704–0.800)) and CH (AUC = 0.750 (95% CI: 0.699–0.802)) in patients with essential hypertension.

The utilization of exercise blood pressure monitoring has been found to be predictive of LVH. Huang *et al*. [84] concluded that adolescents were more accurately diagnosed with hypertension when using exercise blood pressure utilizing submaximal intensity. Several studies have demonstrated that individuals with hypertensive response to exercise had a higher prevalence of LVH, confirmed by an echocardiography [85, 86, 87].

#### 3.4.2 Carotid Intima-Media Thickness (cIMT)

Children with elevated blood pressure, type 1 diabetes, and obesity may have thicker carotid arteries, measured by carotid intima media thickness (cIMT) [88]. Thicker cIMT is associated with metabolic abnormalities, oxidative stress, atherosclerosis, and immune abnormalities, which exacerbates the effect of hypertension [26, 89]. Wang *et al*. [12] found in adults that increased cIMT (β = 0.042 (95% CI: 0.010–0.074), *p* = 0.010) was significantly correlated with increased baPWV compared to normotensive participants. In adolescents, Neuhauser *et al*. [89] concluded the combined effect of hypertension and obesity increased the risk for elevated cIMT (OR: 3.37 (95% CI: 1.41–8.04)). Chung *et al*. [55] identified that children with ambulatory hypertension were at higher risk for elevated cIMT (pooled difference: 0.04 mm (95% CI: 0.02–0.05)) when compared to normotensive children. Furthermore, Obrycki *et al*. [90] found that adolescents with ambulatory hypertension had elevated cIMT z-scores as compared to those who were normotensive. Even with obesity excluded from analysis, patients with ambulatory hypertension were at increased risk for targeted organ damage compared to normotensive adolescents.

Elevated lipid levels may also increase cIMT. Juonala *et al*. [91] demonstrated that children with high non-HDL cholesterol from childhood into adulthood had a significantly increased risk for high cIMT compared to participants with healthy non-HDL cholesterol levels throughout childhood and adulthood. Koskinen *et al*. [92] created a lipid risk model and illustrated that children who presented with prehypertension (OR = 1.4 (95% CI: 1.0–1.9)), hypertension (OR = 1.9 (95% CI: 1.3–2.9)), overweight (OR = 2.0 (95% CI: 1.4–2.9)), obesity (OR = 3.7 (95% CI: 2.0–7.0)), borderline high low-density lipoprotein cholesterol (OR = 1.6 (95% CI: 1.2–2.2)), high low-density lipoprotein cholesterol (OR = 1.6 (95% CI: 1.1–2.1)), and borderline low high-density lipoprotein cholesterol (OR = 1.4 (95% CI: 1.0–1.8)) were more likely to have elevated cIMT. The results of the International Childhood Cardiovascular Cohort (i3C) consortium underscores the importance of identifying cardiovascular risk factors such as hyperlipidemia, elevated BMI, and elevated blood pressure to child and adult hypertension. The study found that children with elevated blood pressure had significantly increased risk for a high cIMT. Additionally, elevated BMI and lipid levels were strongly associated with cardiovascular events and subclinical atherosclerosis [93].

#### 3.4.3 Epicardial Adipose Tissue Thickness and Volume 

Epicardial adipose tissue thickness (EATT) describes the layer of metabolically active tissue on the surface of the myocardium that may be associated with arterial hypertension [94]. Khomova *et al*. [95] found in young adults that EATT was significantly higher in groups with arterial hypertension (7.42 mm (95% CI: 5.22–11.01 mm)) compared to healthy controls (7.17 mm (95% CI: 4.67–9.67 mm)). Schweighofer *et al*. [94] observed that hypertensive children (16.5 ± 1.9 cm^3^) have a significantly higher volume of epicardial adipose tissue (EAT) compared to healthy control children (10.9 ± 1.5 cm^3^). EATT was also significantly greater in hypertensive children (0.8 ± 0.3 cm) compared to healthy control children (0.4 ± 0.1 cm) [94]. Blancas Sánchez *et al*. [96] discovered that elevated EATT was significantly linked to hypertensive children (2.2 ± 0.7 cm) compared to healthy controls (1.8 ± 0.5 cm). Logistical regression analysis showed that age, sex, and BMI seemed to be relevant to EATT in children (adjusted R square = 0.22; *p* < 0.001) [96].

Wacker *et al*. [97] used an MRI-based technique in adolescents to assess EAT volume and showed that obese adolescents (49.6 ± 18.0 cm^3^) compared to lean controls (17.6 ± 6.7 cm^3^) had significantly higher EAT volume and also exhibited significantly increased systolic blood pressure. Reyes *et al*. [98] used echocardiography to calculate EAT volume and thickness and noticed that there was a significant correlation between EAT volume and elevated systolic blood pressure (r = 0.256, *p* = 0.028). The study also determined a cut-off point for EAT thickness, if EAT thickness was >3.17 mm, the chance of having two or more cardiometabolic risk factors was high (OR = 3.1 (95% CI: 1.174–8.022 mm)) [98]. Gustafsson *et al*. [99] yielded similar results, determining that systolic blood pressure was directly associated with EATT (β = 0.18, *p* = 0.02).

### 3.5 Renal and Vasculoar Imaging

#### 3.5.1 Renin-Mediated Hypertension

Up to 10% of pediatric hypertension may be related to suprarenal aortic or renal arterial narrowing [100]. Furthermore, renal artery stenosis and mid-aortic syndrome are significant causes of pediatric hypertension but are under-recognized [101]. Doppler renal sonography may allow for accurate diagnosis of renal artery stenosis, mid-aortic syndrome, and other renal causes in children who present with abnormal blood pressure or urinalysis [100]. Doppler renal sonography has a sensitivity of 90% (95% CI: 68–99%) and specificity of 68% (95% CI: 48–84%) for the detection of aortic and renovascular stenosis [100].

Although Doppler sonography is an effective imaging tool in a majority of patients, it is limited when used on obese patients and may not detect accessory arteries [102]. Given this, other imaging modalities may be used, including contrast-enhanced magnetic resonance angiography (MRA) and computed tomographic angiography (CTA) [102]. MRA and CTA are more accurate imaging techniques when used in patients with normal renal function, yet have a high clinical suspicion of renovascular disease [102]. MRA can be used to detect renal artery stenosis, with several studies reporting specificities and sensitivity ranges of 71 to 100 percent and 88 to 100 percent, respectfully [102]. CTA may also be used for significant stenosis detection, given its specificity of 77 to 98 percent and sensitivity of 88 to 96 percent [102]. In a meta-analysis comparing gadolinium-enhanced MRA versus CTA for renal artery stenosis, both diagnostic imaging modalities were found valuable in detection, with no statistical significance found between both techniques [103].

#### 3.5.2 Endothelial Function

Peripheral arterial tonometry (PAT) is a method that is used to analyze the functional status of microvascular endothelium. Jurko *et al*. [104] found that endothelial function was significantly lower in participants with essential hypertension compared to normotensive (*p* = 0.024) and white-coat hypertensive participants (*p* = 0.032). On the contrary, He *et al*. [105] demonstrated that PAT had weak and mixed correlations with cardiometabolic risks (DBP: r ranged from –0.20 to –0.13, others: |r| < 0.1) as compared to baPWV (|r| ranged from 0.123 to 0.322, *p* < 0.05).

Flow-mediated dilation (FMD) is another method used to analyze endothelial function. Couch *et al*. [106] used FMD to assess adolescents on the Dietary Approaches to Stop Hypertension diet and found greater improvements in FMD (2.5%, *p* = 0.05) and systolic blood pressure (–2.7 mmHg, *p* = 0.03, –0.3 z-score, *p* = 0.03) compared to adolescents on routine care. Jukic *et al*. [107] monitored FMD of the brachial artery in hypertensive and normotensive adolescents and found that FMD was significantly decreased in hypertensive children (8.68% (95% CI: 6.14–12.98)) compared to normotensive children (10.22% (95% CI: 7.00–17.31)).

Retinal vessel diameter may also be an indicator of blood pressure progression in children. Lona *et al*. [24] showed that children with increased systolic or diastolic blood pressure developed narrower central retinal arteriolar (β = –0.154 (95% CI: –0.294 to –0.014 µm per 1 mmHg), *p* = 0.031 and β = –0.02 (95% CI: –0.344 to –0.057 µm per 1 mmHg), *p* = 0.006, respectively). Köchli *et al*. [108] had similar results; retinal arteriolar narrowing were associated with systolic and diastolic blood pressure (systolic blood pressure: –0.63 (95% CI: –0.92 to –0.34); diastolic BP: –0.60 (95% CI: –0.95 to –0.25)).

## 4. Risk Prediction

### 4.1 Arterial Stiffness Risk Prediction Score

Inadome *et al*. [109] developed a risk prediction score for evaluating arterial stiffness that associated increased age, systolic blood pressure, diastolic blood pressure, heart rate, fasting blood sugar, triglyceride, and estimated glomerular filtration rate with impacting arterial stiffness. This risk score yielded an area under the curve (AUC) of 0.68 for men and 0.71 for women. Inadome *et al*. [109] developed a risk prediction equation that yielded an AUC of 0.71 for men and 0.77 for women. Utilizing this information, arterial stiffness can be monitored and utilized as a marker for hypertension, however further validation is required in other ethnic and age groups [109].

### 4.2 Ambulatory Arterial Stiffness Index

Ambulatory arterial stiffness index (AASI), derived from ABPM, is a modality that monitors arterial stiffness for 24 hours and may be used to identify hypertension. Simonetti *et al*. [110] demonstrated that hypertensive children had a higher AASI (AASI = 0.370 (95% CI: 0.250–0.490)) compared to normotensive children (AASI = 0.204 (95% CI: 0.005–0.403)). Furthermore, AASI has been shown to be a strong predictor of systolic and diastolic nocturnal blood pressure dipping (r = –0.369 and –0.305, respectively; *p* < 0.0001). Patients who did not have a drop in nocturnal blood pressure had a significantly higher AASI than patients with a decrease in nocturnal blood pressure, respectively (0.407 ± 0.17 vs. 0.295 ± 0.13; *p* < 0.0001).

### 4.3 Multimodal Composite Risk Models

Mitu *et al*. [111] utilized the “systematic coronary risk evaluation project” (SCORE) to correlate multiple risk factors with subclinical cardiovascular disease in a healthy adult population. The SCORE risk was calculated by assessing cIMT and plaque detection, aortic PWV, echocardiography, LVMI and aortic atheromatosis, and ankle-brachial index. The study found that 60% of participants who were classified to have low to intermediate risk of cardiovascular disease (CVD) exhibited subclinical cardiovascular abnormalities in at least one vascular territory; 27% had two or more markers that were elevated. Both cIMT and aortic PWV were independently associated with elevated SCORE risk while LVMI was directly correlated. This study exhibited the flaws to the SCORE risk assessment, calling for a need for improved risk classification [111].

Chao *et al*. [112] studied the correlation between indicators of vascular aging and hypertensive target organ damage in an adult population. Participants were either classified as having healthy vascular aging (HVA) or non-healthy vascular aging (NHVA). HVA participants have no history of hypertension and a cfPWV ≤7.6 m/s; NHVA participants have a history of hypertension and a cfPWV ≥7.6 m/s. NHVA group showed greater LVMI (108.0 ± 26.4 g/m^2^ vs. 92.3 ± 22.3 g/m^2^, *p* < 0.001), cIMT(0.8 ± 0.1 mm vs. 0.7 ± 0.1 mm, *p* < 0.001), and lower creatinine clearnance compared (88.7 ± 17.2 mL/min/1.73 m^2^ vs.93.4 ± 15.8 mL/min/1.73 m^2^, *p* < 0.001) to HVA. Furthermore, upon multivariate linear regression analysis between vascular aging groups, LVMI was found to be significantly associated NHVA. These findings support the integration of markers such as LVMI, cIMT to develop a comprehensive composite risk model [112].

Recently, Flynn *et al*. [113] conducted the SHIP-AHOY study which assessed 373 adolescents by clinical blood pressure and 24-hour ambulatory blood pressure and were stratified into normal blood pressure, white coat hypertension, ambulatory hypertension and masked hypertension based off auscultatory clinic measurements. Markers of cardiovascular risk the study used included LVMI, E/e’ ratio, ejection fraction, strain, and cfPWV. These metrics were found to be significantly worse in adolescents with ambulatory and masked hypertension even after adjusting for BMI. This study supports the plausibility of a multimodal risk model in adolescents and further study needs to be conducted on developing that model and evaluating its efficacy [113].

## 5. Limitations to Diagnostic Techniques

### 5.1 Challenges to Current Diagnostic Tools for Pediatric Hypertension 

In children, normative values for blood pressure are based on age, sex, height and ethnicity, making it difficult for physicians to memorize and create an accurate diagnosis of hypertension. In pediatrics, choosing a cuff size that is smaller than necessary can also lead to an overestimation in blood pressure. In addition, the cuff needs to be placed over the brachial artery so that the artery collapses and releases when pressure is administered or removed [114]. Furthermore, as postural shifts, arm placement, and back support can affect blood pressure readings, physicians need to limit excessive movement [114]. Additionally, up to 33% of children with blood pressure readings >90th percentile at the beginning of their visit will have normal blood pressure by the end of their visit [115].

### 5.2 Limited Usage of ABPM in Pediatrics

The usage of ABPM in the pediatric population is limited, due to the lack of validated devices for pediatric use and a lack of a standardized protocol for ABPM usage and interpretation [41]. The number of ABPM readings needed in 24 hours to predict cardiovascular outcomes such as arterial stiffening or LVH is still unclear. Additionally, the long-term consequences of white coat hypertension, masked hypertension, isolated nocturnal hypertension, and non-dipping in children are still unknown. There is also limited data regarding the cost-effectiveness of ABPM, particularly in reducing clinic visits, making it difficult for physicians to justify the cost-burden on their patients [41].

### 5.3 Limitations to Arterial Stiffness

Ethnic differences in arterial stiffness may lead to variations in hypertension diagnosis [116]. Mokwatsi *et al*. [117] conducted a study in South Africa with children aged six to eight and noted that PWV and carotid intima-media thickness (cIMT) were higher on average in Black children compared to white children, although carotid ultrasound stiffness indices were similar between the two groups. In the United States, the Minneapolis Children’s Blood Pressure study noted that brachial pulse pressure was higher in Black adolescents after a 9-year follow-up period [118]. Collins *et al*. [119] found that Black adolescents have a higher CAVI, indicating greater arterial stiffness.

## 6. Guidelines for Pediatric Hypertension

### 6.1 Arterial Stiffness

Arterial stiffness is recognized internationally as a marker of early vascular damage; however, several guidelines do not recommend the routine use of this measure in pediatrics. The AAP, ESH, and Korean Working Group of Pediatric Hypertension (KWGPH) are several international guidelines that cite the lack of normative values and investigational nature of arterial stiffness as the reason to not recommend routine arterial stiffens measurements [8, 9, 120]. Research focused networks such i3C and EPOGH utilize arterial stiffness as a measure in order to develop population-based risk stratification and potential normative values, highlighting the growing importance of arterial stiffness in pediatric cardiology [76, 93].

### 6.2 Ambulatory Blood Pressure Monitoring

The usage of ABPM is unanimously recommended by many of the major guidelines as a diagnostic and monitoring tool for pediatric hypertension. The AAP, ESH, and recent KWGPH guidelines regard ABPM as the gold standard for diagnosing hypertension in children. AAP and ESH Furthermore, each guideline provides normative values, adjusted for age, sex, height and weight, that physicians can used to diagnose hypertension. Additionally, ABPM is recommended to be utilized to assist in identifying white coat hypertension and masked hypertension. ABPM is also suggested to be utilized to monitor diurnal blood pressure variability and nocturnal blood pressure dipping patterns [8, 9, 120].

AAP, ESH and KWGPH are in agreeance for the indications to utilize ABPM. AAP specifies to conduct ABPM after 3 elevated office blood pressure readings (≥95th percentile); ABPM should be in use in order to evaluate for white coat hypertension or masked hypertension. ABPM is also recommended for high-risk children, including those with chronic kidney disease, post-coarctation of the aorta repair, diabetes mellitus, solid organ transplant recipients and obese children with co-morbidities. AAP suggests ABPM be performed at initial diagnosis and annually in high risk children. Interpretation of ABPM should be based off normative data and include 24-hour, daytime and nighttime blood pressure, BP load percentages and assessment of nocturnal dipping [8, 9, 120].

### 6.3 Echocardiography

The usage of ECG is supported by AAP, ESH and KWGPH. Both AAP and KWGPH recommend ECG to assess target organ damage such as LVH. LVH is defined as having a LV mass ≥51 g/m^2.7^ for both boys and girls for children older than 8 years. ESH defines LVH as LVMI or RWT ≥95th percentile by age and sex. A repeat ECG should be performed to monitor for improvements to target organ damage at 6 to 12 month intervals. Indications to repeat ECG include persistent hypertension despite treatment, concentric LVH or reduced LV ejection fraction. ECGs should be conducted prior to the administration of pharmaceuticals [8, 9, 120].

## 7. Treatments for Hypertension

**Fig. 3.**
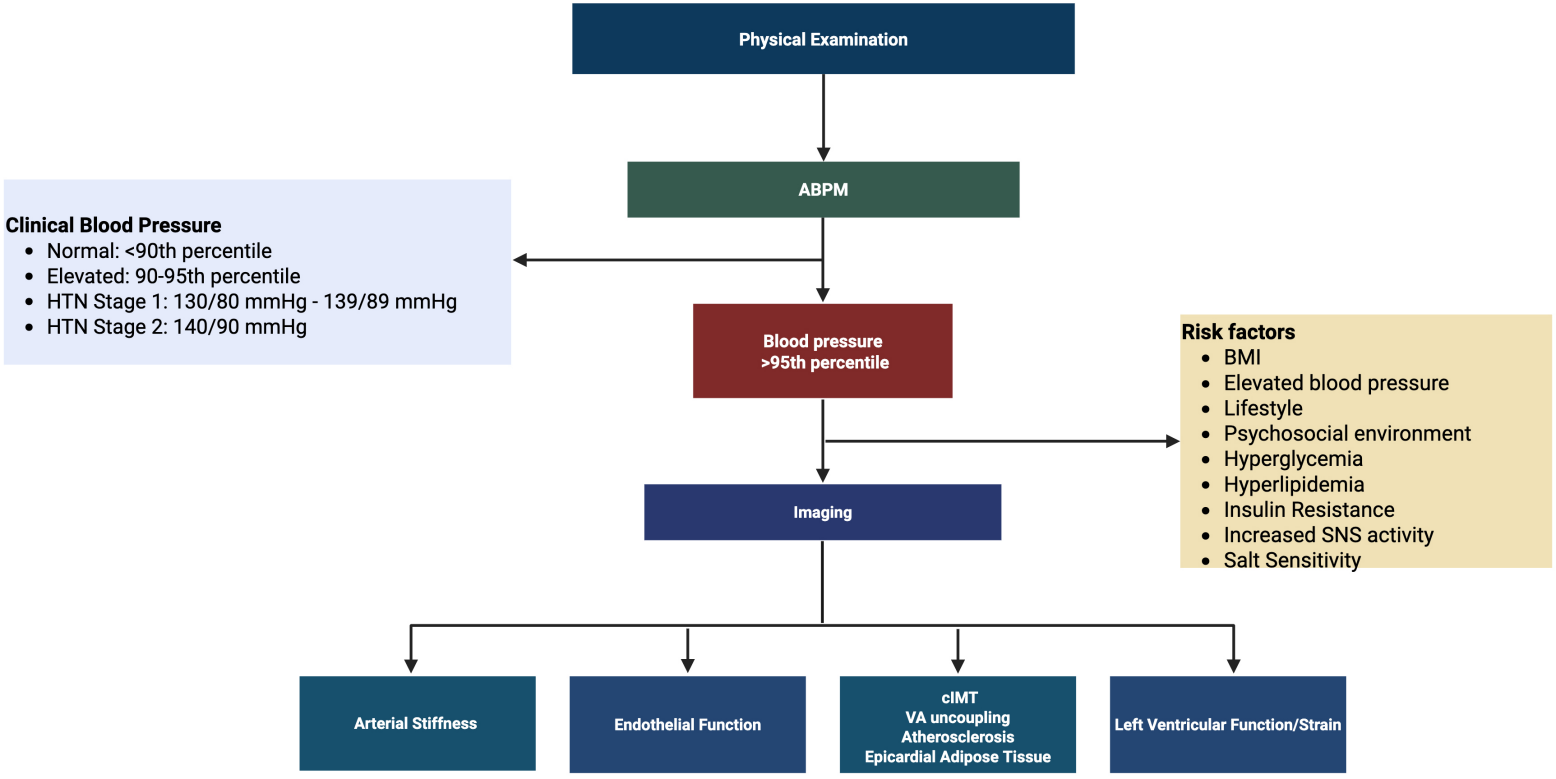
**Hypertension diagnosis algorithm**. Clinical blood pressure guidelines were derived from the American Academy of Pediatrics. Created in BioRender. https://BioRender.com/x97o1c1. Abbreviations: ABP, ambulatory blood pressure; BMI, body mass index; cIMT, carotid intima-media thickness; HTN, hypertension; mmHg, millimeter of mercury; SNS, sympathetic nervous system; VA uncoupling, ventricular arterial uncoupling.

The primary treatment for hypertension includes lifestyle modifications such as increasing physical activity, eating a balanced diet, and having an emotional and social support system (Fig. 3) [121]. Firstline medications include low-dose angiotensin-converting enzyme inhibitors (ACEi), angiotensin receptor blockers (ARB), dihydropyridine calcium channel blockers (CCB), or diuretics. If the low-dose single drug is not effective, a full-dose single drug or low-dose combination drug is recommended. If neither of those treatments is effective, a full-dose combination therapy is recommended as a final line of therapy [121].

## 8. Future Directions

Future directions for diagnosing pediatric hypertension should focus on developing a comprehensive cardiovascular risk score tailored for children and adolescents. This score would ideally integrate dynamic risk factors, including blood pressure and obesity, while exploring the potential benefits of adding genetic markers and social determinants of health [122]. Incorporating this risk score into electronic health records can help facilitate early diagnosis. Similarly, screening tables with diagnostic values of blood pressure in electronic health records can be implemented as well as ensuring accurate staff measurements of blood pressure [123]. Additionally, improving accessibility and tolerability of ABPM in children may improve the accuracy of testing. Future research should investigate the pathophysiological transitions of hypertension from youth to adulthood, the effects of early pharmacological interventions on long-term cardiovascular outcomes, and the efficacy of combination therapies [124].

### 8.1 Artificial Intelligence and Hypertension Monitoring 

Furthermore, with the rise in advanced technology and the rapid development of artificial intelligence, novel technological implementations in cardiology can be more effective in detecting and diagnosing hypertension. Reddy *et al*. [125] recently published the first pediatric-trained AI model that assesses ventricular function. The AI model, titled EchoNet, was able to segment the left ventricle with a Dice similarity of 0.89. EchoNet was also able to estimate ejection fraction with a mean absolute error of 3.66% and can identify pediatric patients with systolic dysfunction with an AUC of 0.95 [125]. Huang *et al*. [126] developed a system driven by AI to monitor nocturnal blood pressure using fiber optic ballistocardiography. This continuous blood pressure monitoring is crucial for screening for hypertension and hypertension management. It is also non-invasive where as current ambulatory blood pressure monitoring systems are, making the AI powered method more favorable [126].

### 8.2 Wearable Devices

Wearable devices health monitoring devices are also becoming high sophisticated with capabilities such as heart rate monitoring and ECG reading. Although more work needs to be done to accurately assess ECG readings while doing exercise, Wang *et al*. [127] was able to show that the Polar H7, Apple watch and Mio Fuse were within 10% of agreeance of an actual ECG reading while at rest. The study was limited due to being a convenience sample in healthy adults, however the study shows that there is health monitoring capabilities to these devices [127]. Lesser known to the masses, the Polar Verity sense and Polar Vantage V2 devices are wearable devices that are claimed to accurately and reliably measure heart rate. Schweizer and Gilgen-Ammann [128] studied both of these devices across a range of physical activities, intensities and wearing positions and found that the Verity sense was highly accurate and reliable, outperforming other wearable heart rate devices and being a potential alternative to ECG-based chest straps. The Vantage V2 had moderate accuracy however it presented with greater variability and lower heart rate readings [128].

## 9. Conclusion

Advancements in cardiovascular risk assessment have significantly improved the early detection and management of hypertension in the pediatric population, incorporating methodologies such as arterial stiffness measurements, ambulatory blood pressure monitoring, endothelial function testing, and genetic markers. These tools provide a more precise understanding of the impact of hypertension on cardiovascular health, allowing for timely interventions that can prevent long-term complications. The integration of lifestyle modifications, alongside emerging diagnostic techniques, offers a comprehensive approach to risk stratification and disease prevention. Future research should continue refining these assessment methods, exploring novel biomarkers, and enhancing predictive models to optimize early intervention strategies. Strengthening these diagnostic frameworks will ultimately contribute to reducing the global burden of hypertension and improving cardiovascular outcomes in young populations.
